# Dynorphin activation of kappa opioid receptor promotes microglial polarization toward M2 phenotype via TLR4/NF-κB pathway

**DOI:** 10.1186/s13578-020-00387-2

**Published:** 2020-03-17

**Authors:** Lin Liu, Yingtong Xu, Hongmei Dai, Shan Tan, Xiao Mao, Zhiheng Chen

**Affiliations:** 1 Department of Pediatrics, The Third Xiangya Hospital, Central South University, No. 138 Tongzipo Road, Yuelu District, ChangshaHunan, 410013 China; 2 Department of Medical Genetics, Maternal and Child Health Hospital of Hunan Province, ChangshaHunan, 410008 China

**Keywords:** Dynorphin, Prodynorphin, Microglia, M1, M2 polarization, TLR4, NF-κb

## Abstract

**Background:**

Microglia-mediated neuroinflammation is associated with epilepsy. Switching microglial polarization from the pro-inflammatory M1 phenotype to the anti-inflammatory M2 phenotype represents a novel therapeutic strategy for mitigating epileptogenesis. We previously found that dynorphins protected against epilepsy via activation of kappa opioid receptor (KOR). Here, this study aims to investigate the role and the mechanism of dynorphin in regulating microglial polarization.

**Methods:**

A pilocarpine-induced rat model of epilepsy was established and lipopolysaccharide (LPS)-activated BV-2 microglial cells were used as an inflammatory model to explore the mechanism of dynorphin regulating microglial polarization.

**Results:**

Overexpression of the dynorphin precursor protein prodynorphin (PDYN) alleviated the pilocarpine-induced neuronal apoptosis, promoted microglial polarization to the M2 phenotype, and inhibited pilocarpine-induced Toll-like receptor 4 (TLR4)/nuclear factor-kappa B (NF-κB) pathway in the hippocampi of epileptic rats. Dynorphin activation of KOR promoted microglial M2 polarization via inhibiting TLR4/NF-κB pathway in LPS-stimulated BV-2 microglial cells. Moreover, dynorphin/KOR regulated microglial M2 polarization inhibited apoptosis of the primary mouse hippocampal neurons.

**Conclusion:**

In conclusion, dynorphin activation of KOR promotes microglia polarization toward M2 phenotype via inhibiting TLR4/NF-κB pathway.

## Background

Epilepsy is a central nervous system (CNS) disorder characterized by spontaneous seizures caused by abnormal electrical activity of large number of neurons in the brain [1]. Neuroinflammation is a significant pathological process involved in all types of CNS damages and disorders [2]. Microglia, the resident macrophage cells of the CNS, has been shown to mediate multiple facets of neuroinflammation [3]. During CNS damage including epilepsy, microglia transform from the resting to an amoebic activated state to combat pathogens or injury [4, 5]. Microglia can be activated in a polarizing manner into a classical phenotype (pro-inflammatory, M1) or an alternative phenotype (anti-inflammatory, M2). The M1 phenotype exhibits strong phagocytic ability and is characterized by increased expression of several proteins including inducible NO synthase (iNOS) and CD16/32, as well as increased release of several pro-inflammatory factors such as interleukin (IL)-1β, IL-6, tumor necrosis factor (TNF)-α [6]. The M2 phenotype is characterized by increased expression of several proteins such as arginase-1 (Arg-1) and mannose receptor (MR/CD206), as well as increased production of anti-inflammatory cytokines IL-4 and IL-10 [1, 3]. Convincing evidence has suggested that the pro- and anti-inflammatory states of microglia indeed determine the outcomes of many CNS diseases, including epilepsy.

Toll-like receptor 4 (TLR4) is primarily expressed in microglia and mediates microglial activation [7]. TLR4 induces activation of nuclear factor-kappa B (NF-κB) via the myeloid differentiation factor 88 (MyD88)/TIR-domain-containing adapter-inducing interferon-β (TRIF) signaling pathway, and ultimately increasing cytokine expression and inflammatory injury [8]. A recent study showed that betaine induced polarization of the microglia to the M2 phenotype by possibly inhibiting the TLR4/NF-κB pathway [3], reinforcing the important role of the TLR4/NF-κB pathway in regulating microglia polarization.

Dynorphins act as endogenous anticonvulsants via activation of kappa opioid receptor (KOR) [9–12]. Dynorphins are a class of opioid peptides that arise from the precursor protein prodynorphin (PDYN). When PDYN is cleaved during processing by proprotein convertase 2 (PC2), several active peptides are released: dynorphin-A, dynorphin-B, α/β-neo-endorphin, and big dynorphin [13, 14]. Our previous study has demonstrated that dynorphin activation of KOR protects against epilepsy and seizure-induced brain injury via PI3K/Akt/Nrf2/HO-1 pathway [15]. It has been shown that KOR activation by U50488H inhibits the TLR4/NF-κB signaling in an ischemia–reperfusion injured rat heart model [16], which suggests that dynorphin activation of KOR may also inhibit TLR4/NF-κB signaling. Therefore, this study aims to investigate whether dynorphin, a KOR activator, regulates TLR4/NF-κB signaling and thus modulates polarization of microglial phenotype.

## Materials and methods

### Animals and ethics statement

Adult male Wistar rats (weight, 200–220 g; years, 8 weeks old) fed a healthy diet under specific pathogen-free conditions were used in our study. The animals were kept at controlled temperatures (25 ± 1 °C) and humidity (50%), with 12 h light–dark cycles. All animal procedures were in compliance with the National Institutes of Health Guidelines for the Care and Use of Laboratory Animals. This study was approved by the Ethics Committee of the Third Xiangya Hospital (Hunan, China).

### Pilocarpine-induced epilepsy in rats and experimental groups

Rats were randomly divided into four groups (n = 8/group): (1) Control group, (2) epileptic model group, (3) LV-NC group, and (4) LV-PDYN group. The epilepsy was induced by pilocarpine injection as described in our previous study [15]. Briefly, the rats in the epilepsy model group were injected intraperitoneally (i.p.) with lithium chloride (127 mg/kg; Sigma-Aldrich, USA) 18 h prior to the first administration of pilocarpine (30 mg/kg, i.p., Sigma-Aldrich). Pilocarpine (10 mg/kg, i.p.) was then repeatedly injected every 30 min until the rats developed seizures. Rats in the control group received an intraperitoneal injection with isovolumic normal saline instead of pilocarpine. One hour after the onset of the status epileptics, rats were injected with diazepam (10 mg/kg, i.p.) to terminate seizures.

Rats in the LV-NC group and LV-PDYN group received a stereotaxic intra-hippocampus injection of LV-NC (a negative control lentiviral vector; GeneChem, Shanghai, China) and LV-PDYN (a lentiviral vector stably overexpressing PDYN, GeneChem) respectively as previously described before the establishment of the epilepsy model [17]. The coding sequence of PDYN was amplified by RT-PCR and ligated them into the pGC-FU plasmid to produce pGC-FU-PDYN -green fluorescent protein (GFP) (LV-PDYN). A lentiviral vector expressing GFP alone was chosen as a negative control (LV-NC). Briefly, before the pilocarpine-induced seizure, rats were deeply anesthetized and the head was fixed in a stereotaxic frame, after which 5 μL of LV-PDYN and LV-NC were infused through a glass pipette (0.2 µL/min) bilaterally in the dorsal hippocampus of epileptic rats. The site of virus injection was as follows: anterior–posterior—3.3 mm, medial–lateral ± 1.8 mm, and dorsal–ventral—2.6 mm. The titer of the lentivirus was 2 × 10^9^ TU/mL. The pipette was placed at least 10 min after injection to prevent reflux. After lentivirus injection, all of rats were kept in a standard environment for 1 week, and then given pilocarpine to observe acute seizure features. Animals were sacrificed under deep anesthesia 24 h after status epilepticus. The hippocampus was removed and prepared for further analysis.

### TdT-mediated dUTP Nick-End Labeling (TUNEL) staining

TUNEL staining was performed to analyze the apoptosis in rat hippocampus. Briefly, the 5 μm-thick sections were dewaxed, rehydrated, and incubated with proteinase K (20 µg/mL) at 37 °C for 20 min. After that, the sections were washed with phosphate-buffered saline (PBS) twice and then incubated with the TUNEL reaction mixture provided by the in situ cell death detection kit (Roche, Switzerland). After washing with PBS three times, the sections were stained with diaminobenzidine (Sigma-Aldrich) for 10 min. Finally, the sections were sealed and images were acquired by an Olympus microscope. TUNEL positive cells (brown) was counted and compared with the total cells using Image-Pro Plus 6.0 software and expressed as a percentage.

### Immunofluorescent staining

The sections were dewaxed, rehydrated, and then immersed into citrate buffer for antigen repair. After that, the sections were blocked with 2% bovine serum albumin and then incubated overnight at 4 °C with the following primary antibodies: anti-iNOS (1:200; Invitrogen, USA), anti-ionized calcium binding adaptor molecule-1 (Iba-1) (1:100, Abcam, USA), anti-Arg-1 (1:200, Santa Cruz Biotechnology, USA), followed by incubation with the secondary antibodies Alex Fluor® 488-labeled secondary antibody (green; 1:1000; Abcam), Alex Fluor® 647-labeled secondary antibody (red; 1:1000; Abcam) and Hoechst (blue; 1:1000) for 1 h at room temperature. Slides were observed by a fluorescent microscope (Olympus IX-71) equipped with a Canon EOS digital camera. The iNOS, Arg-1, and Iba-1 positive cells were separately counted using Image J software.

### Cell culture and treatment

The mouse microglial cell line (BV-2) was obtained from BeNa Culture Collection (China). Cells were maintained in Dulbecco's modified Eagle's medium (DMEM, Gibco, USA) containing 10% fetal bovine serum (FBS, Gibco) in a humidified atmosphere of 95% air and 5% CO_2_. Dynorphin-A was used as a KOR agonist and GNTI as a KOR antagonist. Lipopolysaccharide (LPS) was used to activate BV-2 microglial cells. Dynorphin-A and LPS were purchased from Sigma-Aldrich. GNTI was from R&D Systems (USA). To investigate the role of KOR activation in regulating BV-2 microglia polarization, BV-2 cells were pretreated with dynorphin-A or GNTI for 1 h, both alone and in combination, and then treated with or without LPS (1 µg/mL) for 24 h.

The primary mouse hippocampal neurons (Thermo Fisher Scientific) were cultured in Complete Neurobasal® supplemented with GlutaMAX™-I and B-27® (Thermo Fisher Scientific). To investigate the effect of dynorphin/KOR-regulated microglia polarization on neuronal viability and apoptosis, the conditioned medium of BV-2 cells in the groups of Control, LPS, LPS + Dynorphin-A, LPS + GNT1, and LPS + Dynorphin-A + GNT1 were collected and co-cultured with the primary mouse hippocampal neurons. The neuronal viability was examined by CCK-8 assay. The neuronal apoptosis was examined by flow cytometry with Annexin-V-FITC/PI dual-labeling. The protein levels of apoptosis-related caspase-3, Bax, and Bcl-2 in the primary mouse hippocampal neurons were examined by western blot.

### Quantitative real-time PCR (qRT-PCR)

Total RNA was extracted from rat hippocampus or cultured BV-2 cells using TRIzol reagent (Invitrogen), and reverse-transcribed to cDNA using a Revert Aid First Strand cDNA Synthesis Kit. The mRNA expression of PDYN was detected using Mx3000p (Stratagene, USA), according to methods described previously [18]. The relative quantification was calculated using the 2^−ΔΔct^ method. GAPDH was used as the internal control.

### Western blot

Briefly, rat hippocampus tissues and cultured cells were lysed in RIPA lysis buffer (Beyotime, China). After centrifuging at 14,000*g* at 4 °C for 30 min, the supernatant was collected and separated by 10% SDS-PAGE gels and transferred onto PVDF membrane (Millipore, USA). After being blocked with 5% non-fat milk, the membrane was incubated overnight at 4 °C with the following primary antibodies: anti-PDYN (1:1000; Abcam), anti-TLR4 (1:500; Abcam), anti-IκB-α (1:1000, Proteintech, USA), anti-phosphor-(p)-IκB-α (1:1000, Proteintech), anti-NF-κB p65(1:1000, Proteintech), anti-p-NF-κB p65 (1:1000, Proteintech), anti-caspase-3 (1:1000; Abcam), anti-Bax (1:1000, Abcam), anti-Bcl-2 (1:1000; Abcam), followed by horseradish peroxidase-labeled secondary antibodies (1:5000; Proteintech) for 1 h. Quantity One (Bio-Rad Laboratories, USA) software was used to analyze the results. β-actin and α-tubulin were used as the internal references.

### Quantification of cytokine levels

The levels of IL-1β, IL-6, IL-4, and IL-10 were measured using their commercial enzyme-linked immunosorbent assay (ELISA) kits (R&D Systems) according to the manufacturer’s instructions.

### Flow cytometry analysis

The percentage of M1-related CD16/32 positive cells and M2-related CD206 positive cells were examined by flow cytometry. Briefly, the BV-2 cells were collected, washed with PBS and adjusted to 1 × 10^6^ cells/mL. Afterward, PE-conjugated anti-CD16/32 (BioLegend, USA) and FITC-conjugated anti-CD206 (BioLegend) were added. After 1 h of incubation at 4 °C in the dark, the cells were washed with PBS and resuspended in 500 μL of PBS solution. The mixtures were analyzed with a BD Accuri™ C6 flow cytometer (BD Biosciences, USA).

To evaluate cell apoptosis in the primary mouse hippocampal neurons, flow cytometry analysis was performed using the FITC-conjugated Annexin V apoptosis detection kit (BD Pharmingen, USA) according to the manufacturer’s instructions. The data were analyzed using FlowJo software (Tree Star, USA).

### Cell viability assay

Cell viability assay was performed using the Cell Counting Kit‐8 (CCK‐8; Beyotime). Briefly, the primary mouse hippocampal neurons were seeded in the 96‐well plates and were co-cultured with the conditioned medium of BV-2 cells in the groups of Control, LPS, LPS + Dynorphin-A, LPS + GNT1, and LPS + Dynorphin-A + GNT1, then the OD_450_ values were measured using the Fluoroskan Ascent Fluorometer (Thermo Fisher Scientific).

### Statistical analysis

All statistical analyses were performed using SPSS statistical software package standard version 16.0 (SPSS, Inc., USA). The data are presented as the mean ± standard deviation (SD) from three independent experiments. Group statistical significance was assessed using Student’s t-test for comparison of two groups, one-way ANOVA for three or more groups. p < 0.05 was considered statistically significant.

## Results

### PDYN overexpression alleviated pilocarpine-induced neuronal apoptosis in rats

**Fig. 1:**
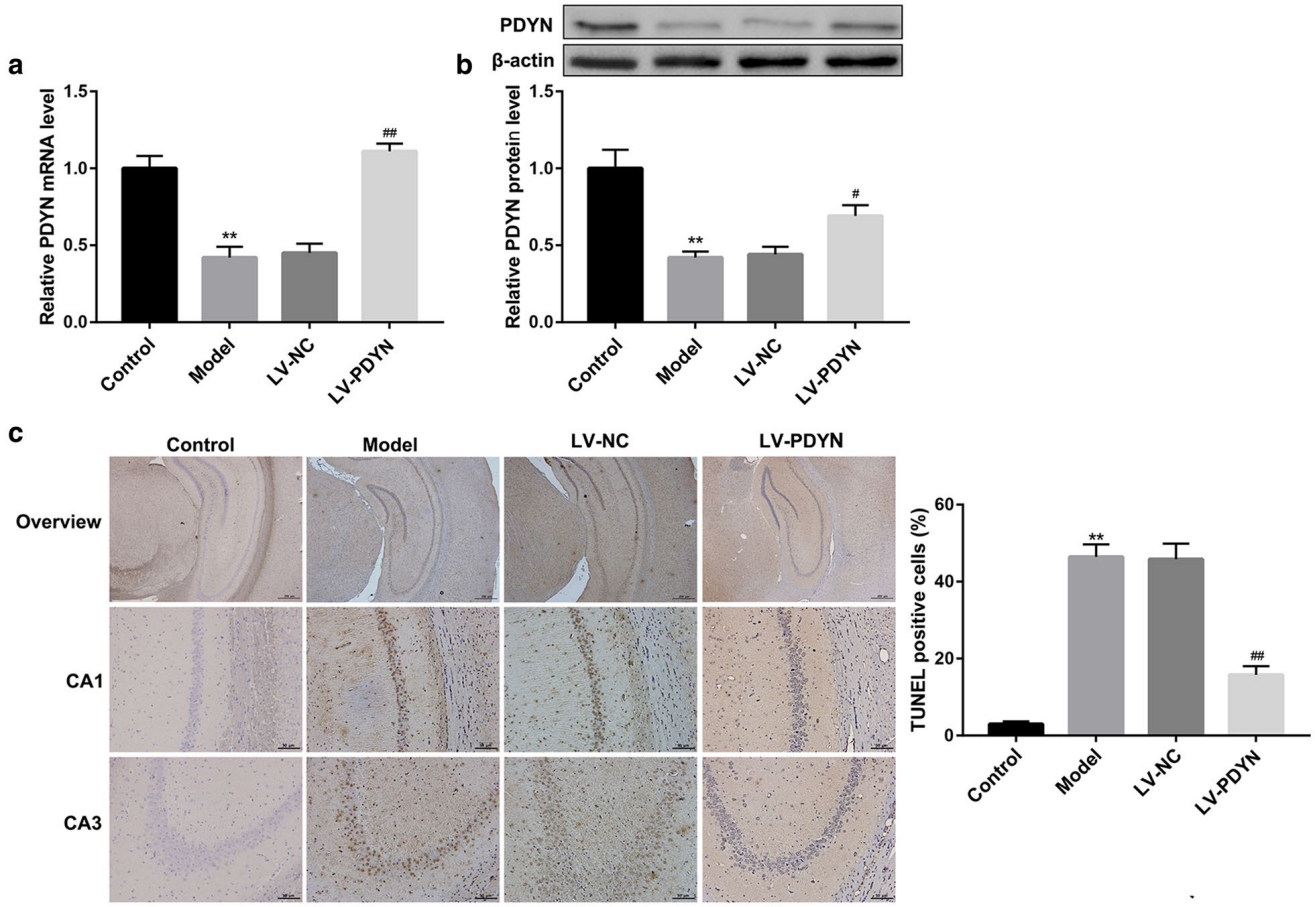
PDYN overexpression alleviated pilocarpine-induced neuronal apoptosis in rats. Rats were randomly divided into four groups: Control, epileptic model, LV-NC, and LV-PDYN group. **a** qRT-PCR analysis of PDYN mRNA expression and **b** western blot analysis of PDYN protein level in rat hippocampus. **c** Representative TUNEL staining images of rat hippocampus and percentage of TUNEL-positive cells. n = 8/group. **p < 0.01, vs. Control group, ^#^p < 0.05, ^##^p < 0.01, vs. LV-NC group

We first examined PDYN expression in the hippocampus of epileptic rats using qRT-PCR and western blot. The PDYN mRNA (Fig. 1a) and protein (Fig. 1b) levels were notably decreased in the Model group in comparison with the control group. To explore the in vivo role of PDYN in neuronal apoptosis in a rat model of pilocarpine-induced epilepsy, we injected PDYN-overexpressing lentiviruses and its control into the dorsal hippocampus of epileptic rats. As expected, compared with the LV-NC group, LV-PDYN administration significantly restored the reduction of PDYN mRNA (Fig. 1a) and protein (Fig. 1b) levels in the epilepsy model rats. Furthermore, TUNEL staining demonstrated that intra-hippocampus injection of LV-PDYN markedly attenuated the pilocarpine-induced hippocampal neuronal apoptosis (Fig. 1c). These data indicated that PDYN overexpression alleviated pilocarpine-induced neuronal apoptosis in rats.

### PDYN overexpression promoted microglial polarization to the M2 phenotype in a rat model of epilepsy

**Fig. 2:**
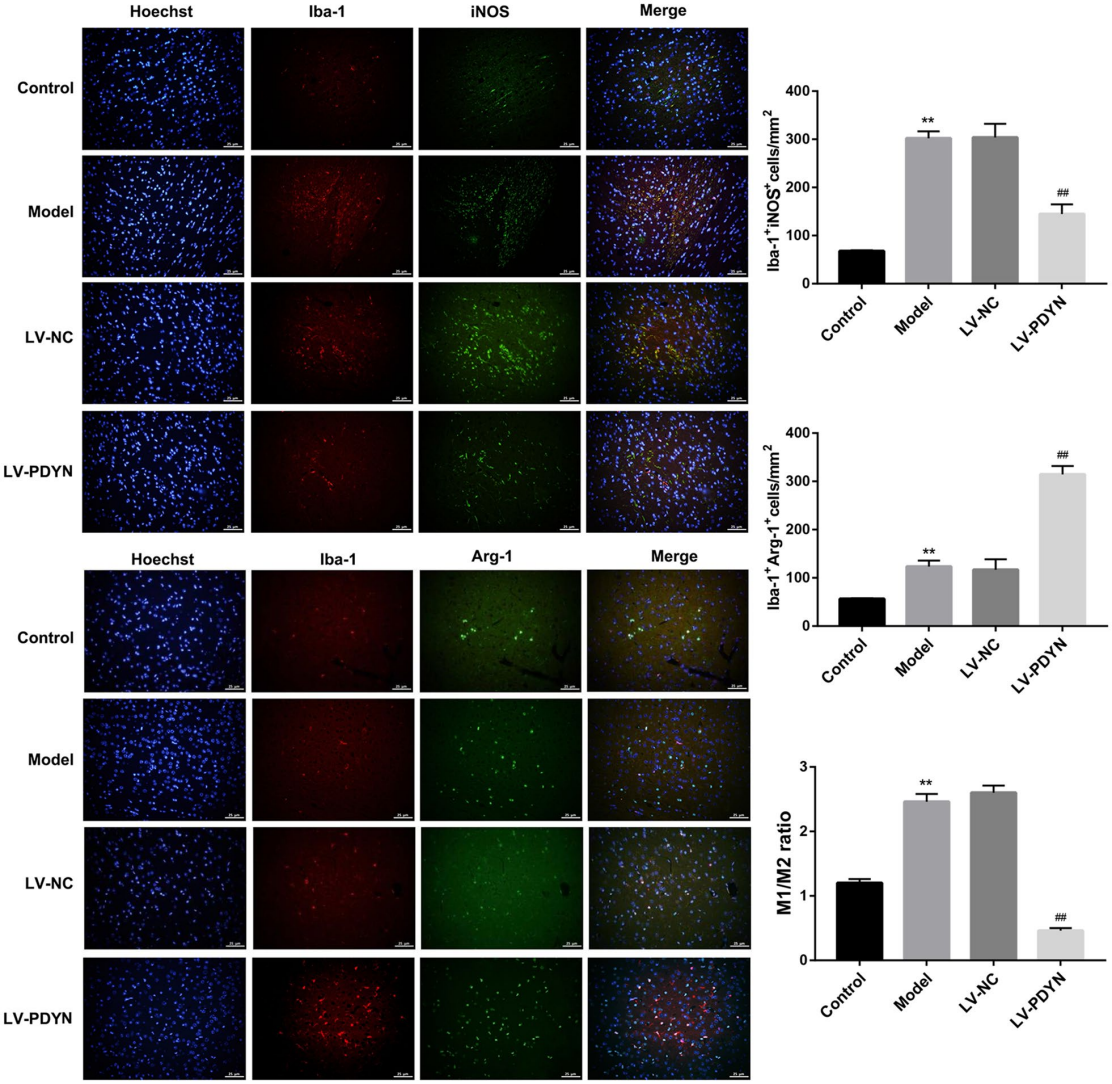
PDYN overexpression promoted microglial polarization to the M2 phenotype in a rat model of epilepsy. Representative immunofluorescence images in rat hippocampus sections. Hoechst (nuclei, blue), total microglia (Iba-1; red), M1-microglia phenotype (iNOS; green), and M2-microglia phenotype (Arg-1, green). Quantitative analysis for the number of Iba-1^+^/iNOS^+^ cells (M1-phenotype markers), and for the number of Iba1^+^/Arg-1^+^ cells (M2-phenotype markers). n = 8/group. **p < 0.01, vs. Control group, ^##^p < 0.01, vs. LV-NC group

Switching microglial polarization from the M1 phenotype to the M2 phenotype represents a novel therapeutic strategy for mitigating epileptogenesis [1, 19]. To explore whether LV-PDYN administration alters microglia polarization, we used antibodies specific to Iba-1 (a marker of microglial activation) and iNOS (a M1 marker of microglia) to identify M1, and antibodies specific to Iba-1 and Arg-1 (a M2 marker of microglia) to identify M2. Results of immunofluorescent staining showed that pilocarpine caused a significant increase in the number of Iba-1^+^iNOS^+^ cells (M1) and Iba-1^+^Arg-1^+^ cells (M2) in rat hippocampus, indicating microglial activation in the rat model of epilepsy. Furthermore, administration of LV-PDYN resulted in a considerable increase in the number of Iba-1^+^Arg-1^+^ cells (M2) but a notable decrease in that of Iba-1^+^iNOS^+^ cells (M1) and ratio of M1/M2 (Fig. 2).

**Fig. 3:**
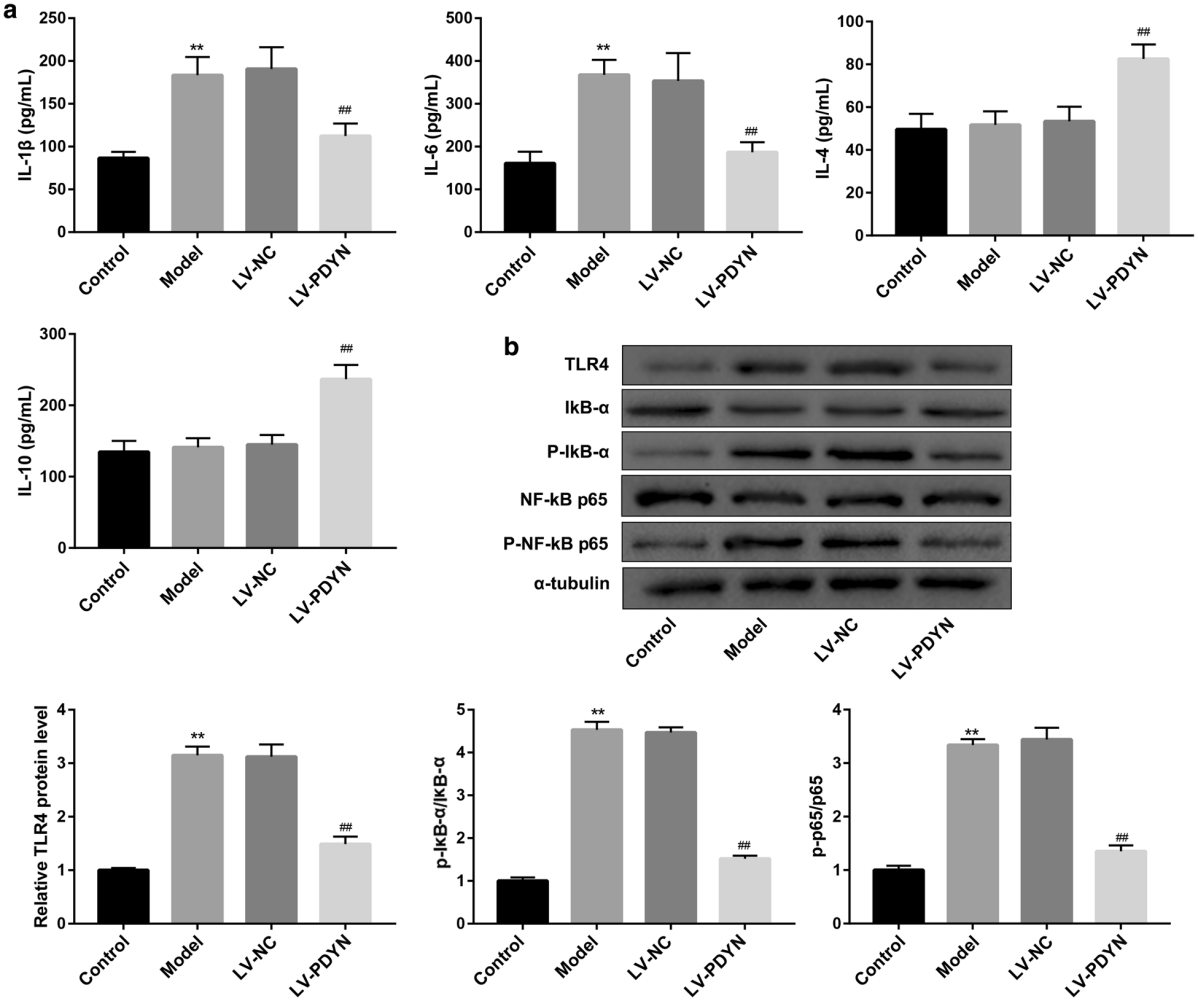
PDYN overexpression inhibited pilocarpine-induced TLR4/NF-κB pathway in a rat model of epilepsy. **a** The serum levels of M1-related pro-inflammatory cytokines (IL-1β and IL-6) and M2-related cytokines (IL-4 and IL-10) in rats were examined by ELISA. **b** The protein levels of TLR4, IκB-α, p-IκB-α, NF-κB p65, and p-NF-κB p65 were examined by western blot. n = 8/group. **p < 0.01, vs. Control group, ^##^p < 0.01, vs. LV-NC group

To corroborate the histological findings, we qualified the serum levels of M1-related pro-inflammatory cytokines (IL-1β and IL-6) and M2-related cytokines (IL-4 and IL-10) in rats. As shown in Fig. 3a, PDYN overexpression notably decreased the pilocarpine-induced levels of M1-related cytokines (IL-1β and IL-6). On the other hand, serum levels of M2-related cytokines (IL-4 and IL-10) were not changed by pilocarpine. However, intra-hippocampus injection of LV-PDYN markedly elevated levels of IL-4 and IL-10. Together, these results suggested that PDYN overexpression shifted pilocarpine-induced inflammatory M1 microglia toward anti-inflammatory M2 microglia.

### PDYN overexpression inhibited pilocarpine-induced TLR4/NF-κB pathway in a rat model of epilepsy

The TLR4/NF-κB pathway plays an important role in regulating microglia polarization [3]. To clarify whether PDYN regulates microglial polarization via TLR4/NF-κB pathway, we examined protein levels of TLR4/NF-κB pathway-related proteins. Western blot analysis revealed that the epilepsy model rats exhibited higher protein level of TLR4 and phosphorylation of IκB-α and NF-κΒ p65 when compared with the control group, indicating activation of TLR4/NF-κB pathway. However, administration of LV-PDYN significantly inhibited pilocarpine-induced activation of TLR4/NF-κB pathway (Fig. 3b).

### Dynorphin activation of KOR promoted microglial M2 polarization via inhibiting TLR4/NF-κB pathway in LPS-stimulated BV-2 microglial cells

**Fig. 4:**
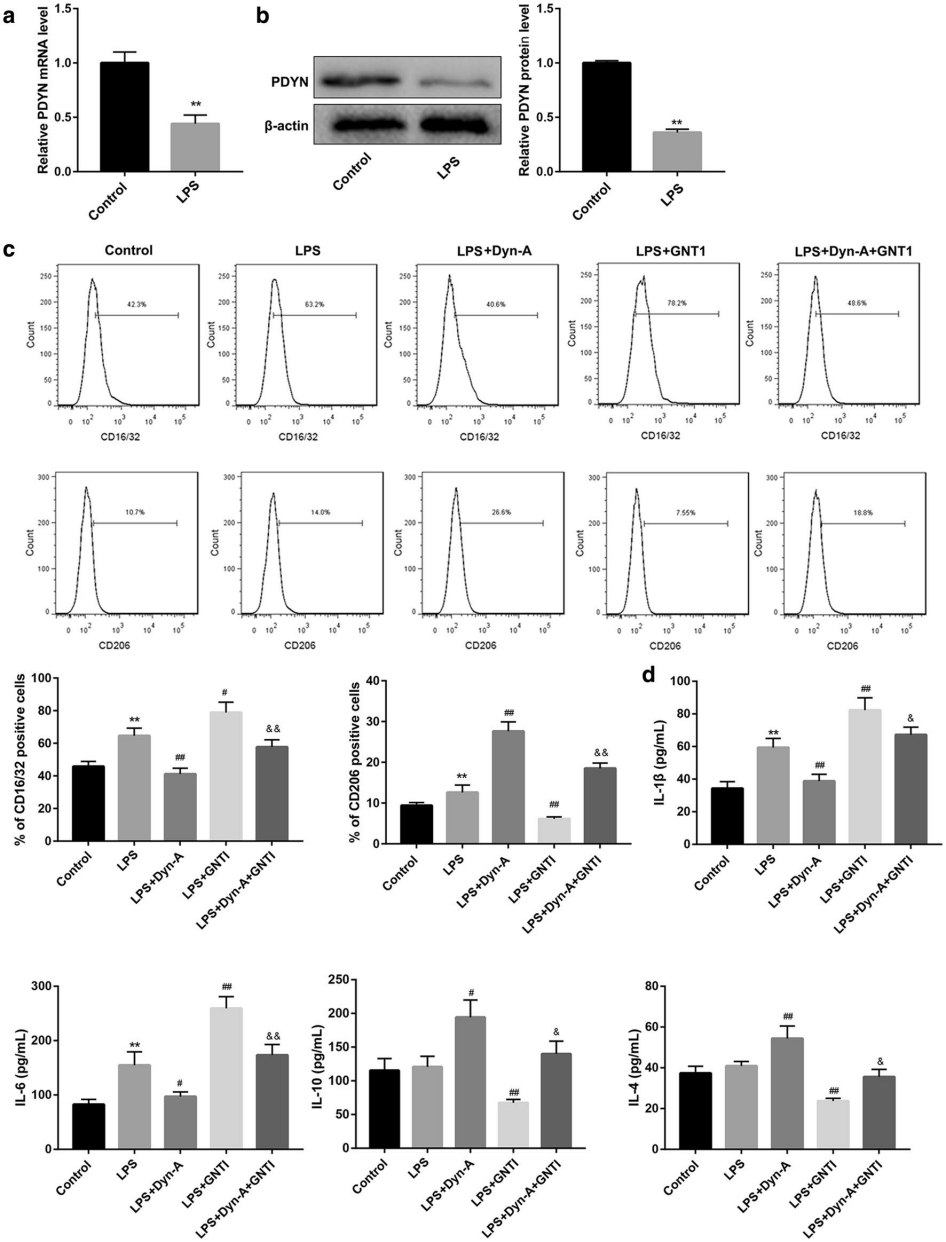
Dynorphin activation of KOR promoted microglial M2 polarization in LPS-stimulated BV-2 microglial cells. **a** qRT-PCR analysis of PDYN mRNA expression and **b** western blot analysis of PDYN protein level in BV-2 microglial cells stimulated without or with LPS (1 µg/mL, 24 h). **c** Determination of CD16/32 and CD206 expression by flow cytometry and **d** levels of M1-related cytokines (IL-1β and IL-6) and M2-related cytokines (IL-4 and IL-10) by ELISA in BV-2 cells in the groups of Control, LPS, LPS + Dyn-A, LPS + GNT1, and LPS + Dyn-A + GNT1. N = 3. Dyn-A, Dynorphin-A. **p < 0.01, vs. Control group, ^#^p < 0.05, ^##^p < 0.01, vs. LPS group; ^&^p < 0.05, ^&&^p < 0.01, vs. LPS + Dyn-A group

To further verify whether PDYN regulates microglial polarization via inhibiting TLR4/NF-κB pathway, we used LPS-activated BV-2 microglial cells as an inflammatory model. PDYN mRNA (Fig. 4a) and protein levels (Fig. 4b) were significantly decreased after LPS stimulation. Since CD16/32 and CD206 are specific M1 and M2 polarization markers, respectively, we detected expression of CD16/32 and CD206 by flow cytometry and found that LPS stimulation significantly increased CD16/32 and CD206 expression. Furthermore, LPS stimulation caused increased production of M1-related cytokines (IL-1β and IL-6) but had no significant effect on that of M2-related cytokines (IL-4 and IL-10). Importantly, treatment with dynorphin-A, an endogenous KOR agonist, inhibited the LPS-induced expression of CD16/32 and production of M1-related cytokines (IL-1β and IL-6). However, dynorphin-A increased expression of CD206 and production of M2-related cytokines (IL-4 and IL-10) in LPS-stimulated BV-2 cells. In contrast with dynorphin-A, the KOR-specific antagonist GNTI yielded the opposite effects and effectively abrogated the dynorphin-A-mediated promotion of BV-2 microglial polarization to the M2 phenotype (Fig. 4c, d).

**Fig. 5:**
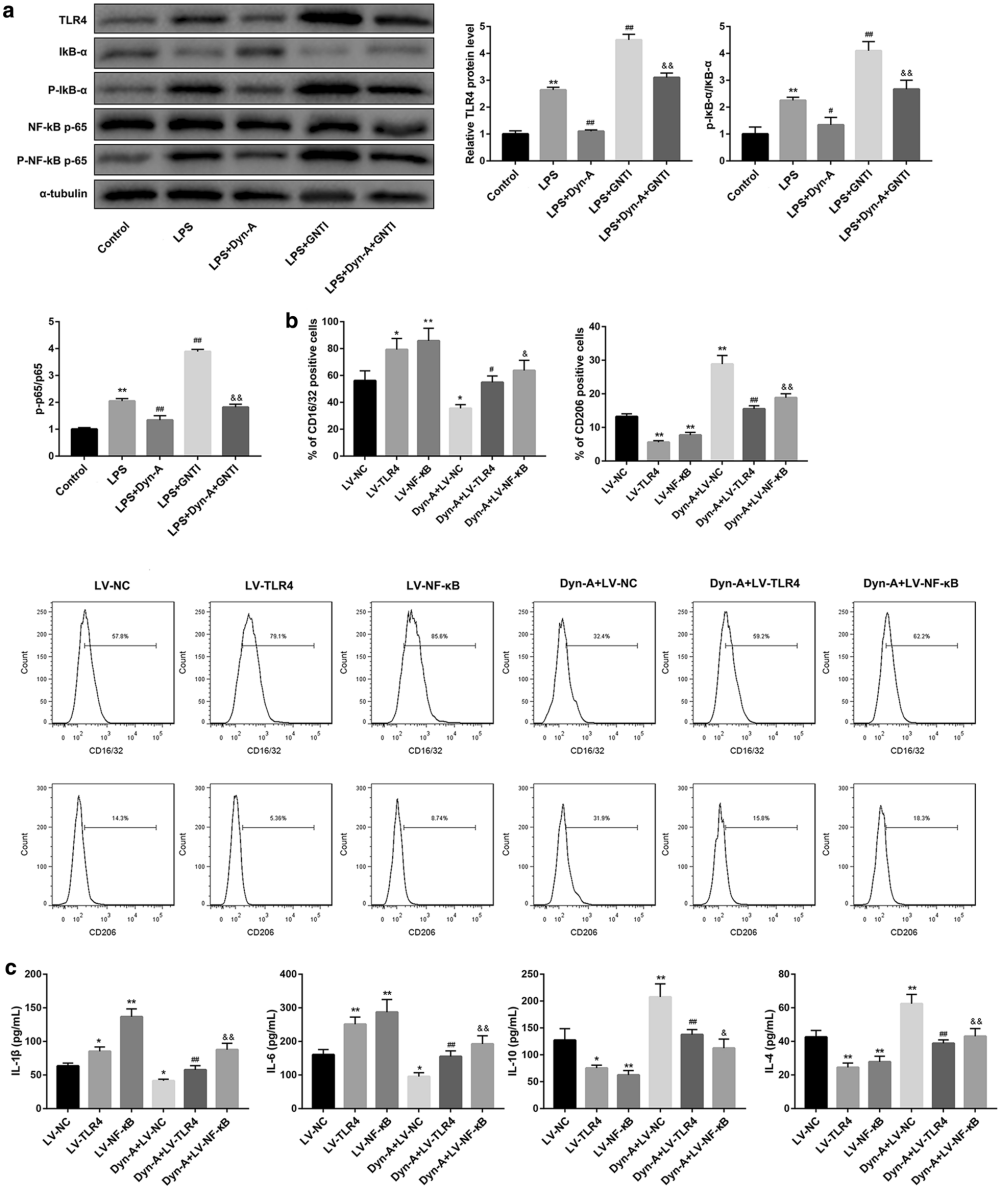
Dynorphin activation of KOR promoted microglial M2 polarization via inhibiting TLR4/NF-κB pathway in LPS-stimulated BV-2 microglial cells. **a** Determination of TLR4, IκB-α, p-IκB-α, NF-κB p65, and p-NF-κB p65 protein levels by western blot in BV-2 cells in the groups of Control, LPS, LPS + Dyn-A, LPS + GNT1, and LPS + Dyn-A + GNT1. **b** Determination of CD16/32 and CD206 expression by flow cytometry and **c** levels of M1-related cytokines (IL-1β and IL-6) and M2-related cytokines (IL-4 and IL-10) by ELISA in LPS-stimulated BV-2 cells in the groups of LV-NC, LV-TLR4, LV-NF-κB, Dyn-A + LV-NC, Dyn-A + LV-TLR4, and Dyn-A + LV-NF-κB. N = 3. Dyn-A, Dynorphin-A. *p < 0.05, **p < 0.01, vs. Control or NC group, ^#^p < 0.05, ^##^p < 0.01, vs. LPS or LV-TLR4 group; ^&^p < 0.05, ^&&^p < 0.01, vs. LPS + Dyn-A or LV-NF-κB group

Moreover, western blot analysis showed that dynorphin-A activation of KOR inhibited the LPS-induced TLR4/NF-κB pathway, which can be abolished by GNTI inhibition of KOR (Fig. 5a). Of note, in contrast with dynorphin-A, overexpression of TLR4 and NF-κB caused a significant elevation of expression of M1-related CD16/32, IL-1β and IL-6, but a reduction of M2-related CD206, IL-4, and IL-10 in LPS-stimulated BV-2 microglial cells (Fig. 5b, c). Moreover, overexpression of TLR4 and NF-κB significantly abrogated the dynorphin-A-mediated promotion of BV-2 microglial M2 polarization (Fig. 5b, c). Taken together, these data suggested that dynorphin activation of KOR promoted microglial M2 polarization via inhibiting TLR4/NF-κB pathway.

### Dynorphin/KOR regulated microglial M2 polarization inhibited neuronal apoptosis

**Fig. 6:**
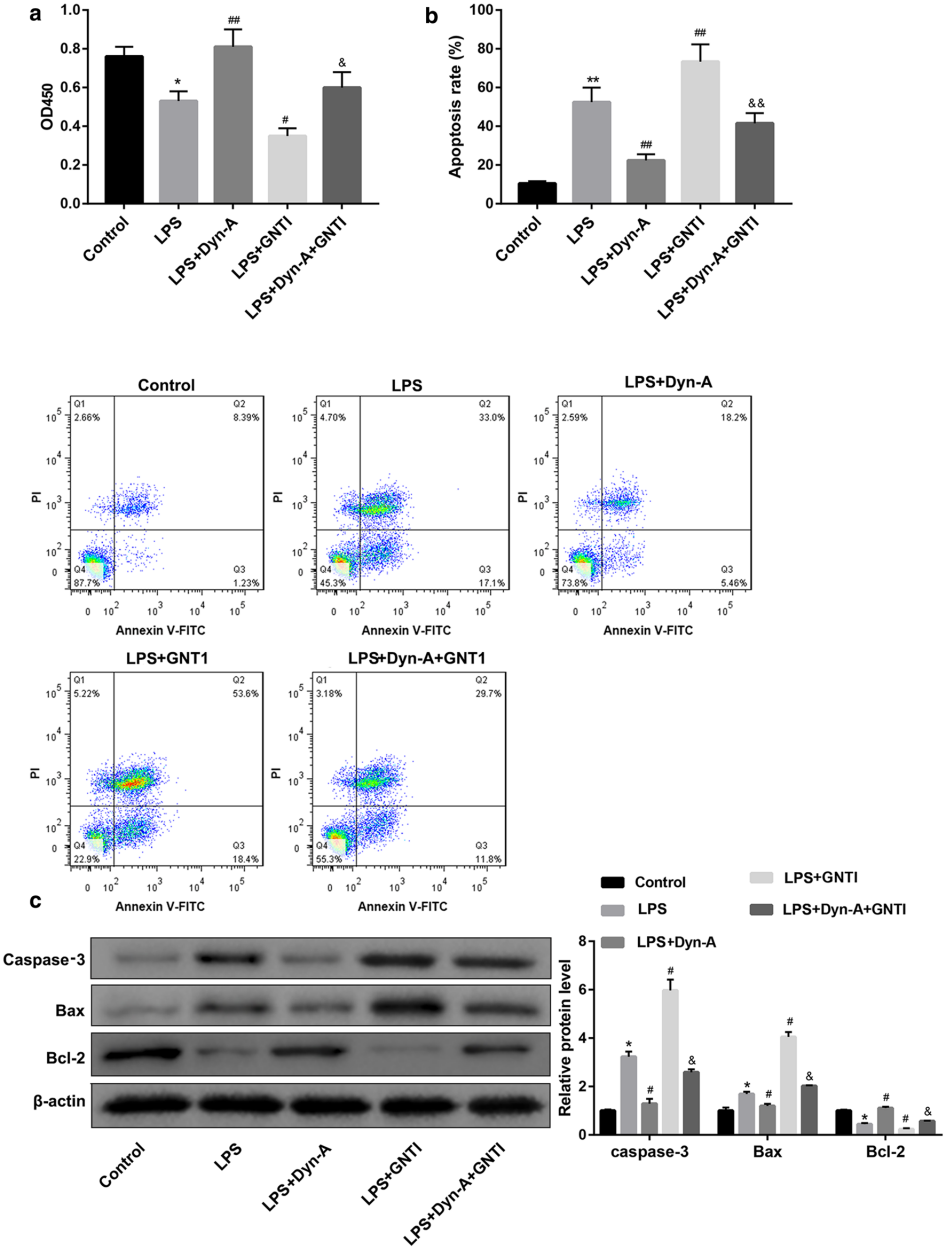
Dynorphin/KOR regulated microglial M2 polarization inhibited neuronal apoptosis. The conditioned medium of BV-2 cells in the groups of Control, LPS, LPS + Dynorphin-A, LPS + GNT1, and LPS + Dynorphin-A + GNT1 were collected and co-cultured with the primary mouse hippocampal neurons. **a** The neuronal viability was examined by CCK-8 assay. **b** The neuronal apoptosis was examined by flow cytometry with Annexin-V-FITC/PI dual-labeling. **c** The protein levels of apoptosis-related caspase-3, Bax, and Bcl-2 in the primary mouse hippocampal neurons were examined by western blot. *p < 0.05, **p < 0.01, vs. Control group, ^#^p < 0.05, ^##^p < 0.01, vs. LPS group; ^&^p < 0.05, ^&&^p < 0.01, vs. LPS + Dyn-A group

To investigate the effect of dynorphin/KOR-regulated microglia polarization on neuronal viability and apoptosis, the conditioned medium of BV-2 cells in the groups of Control, LPS, LPS + Dynorphin-A, LPS + GNT1, and LPS + Dynorphin-A + GNT1 were collected and co-cultured with the primary mouse hippocampal neurons. Data revealed that the neurons co-cultured with the medium from LPS + Dynorphin-A-treated BV-2 cells exhibited higher cell viability but lower cell apoptosis rate, in comparison with those in the LPS group (Fig. 6a, b). Western blot analysis consolidated the results of flow cytometry, as evidenced by a reduction of pro-apoptotic caspase-3 and Bax and elevation of anti-apoptotic Bcl-2 in the LPS + Dynorphin-A group when compared with the LPS group (Fig. 6c). These results suggested that dynorphin/KOR regulated microglial M2 polarization promoted neuronal viability and inhibited neuronal apoptosis. Moreover, the effect of dynorphin-A was compromised by GNTI inhibition of KOR (Fig. 6a–c).

## Discussion

Microglia mediates multiple facets of neuroinflammation [3], which is a significant pathological process involved in all types of CNS disorders including epilepsy [2]. Switching microglial polarization from the pro-inflammatory M1 phenotype to the anti-inflammatory M2 phenotype has been suggested as a novel therapeutic strategy for alleviating epileptogenesis [1, 19]. In a previous study from our laboratory, we demonstrated that the dynorphin activation of KOR not only alleviated pilocarpine-induced epilepsy and neuronal apoptosis in rats, but also in vitro seizure-like neuron injury [15]. Based on these findings, the present study was designed to investigate the role and the mechanism of dynorphin in regulating microglial polarization. Here, we further demonstrate that dynorphin activation of KOR promotes microglia polarization toward M2 phenotype via TLR4/NF-κB pathway. These findings provide new insights into the mechanism of dynorphin in protecting against epilepsy.

We examined the microglia state in the pilocarpine-induced epilepsy rats and found that these epilepsy model rats exhibited a significant increase in the number of Iba-1^+^iNOS^+^ cells (M1) and Iba-1^+^Arg-1^+^ cells (M2) in rat hippocampus when compared with the control rats, indicating microglial activation. Consistent with our results, there are many studies showing that microglia can be activated to combat pathogens or injury during epilepsy [4, 5]. Activated microglia has been observed in the brains of patients with epilepsy-related diseases and animal models of epilepsy [1, 19, 20]. Contradictory to mainstream theories, Zhao et al. [21] recently demonstrated that non-inflammatory changes of microglia are sufficient to cause epilepsy, revealing an epileptogenic mechanism that is independent of the microglial inflammatory response. The reasons for this discrepancy in findings are unclear but could relate to differences in models and detection methods in different studies. Anyway, these findings further cement the notion that microglia are a potential therapeutic target for epilepsy prevention.

In the current investigation, we also analyzed microglial polarization after epilepsy. Our findings indicated that the M1 phenotype of microglia was activated during epilepsy. Microglia can be activated into a pro- or anti-inflammatory state, defined as the M1 and M2 phenotypes, respectively. Li et al. [19] performed flow cytometry and found that M1 and M2 microglia underwent variations throughout the stages of epileptogenesis in a mouse model of pilocarpine-induced epilepsy. Liu et al. [1] observed steadily increasing M1 microglia/macrophages after status epilepticus in the hippocampi of mice, whereas the M2 marker Arg-1 was localized mainly in astrocytes rather than in microglia/macrophages. Recent studies suggest that activated microglia exert different effects on brain function depending on the phase and severity of epileptogenesis [22]. Short-term microglial activation may be beneficial [23], whereas chronic microglial activation may be detrimental [24] for the pathogenesis of epilepsy. Augmented M1 polarization of microglia persists into the chronic phase after status epilepticus. Thus, we speculate that microglial M1 polarization may be involved in the deleterious microenvironment in the brain.

We then investigated the role of dynorphin and its precursor protein PDYN in modulating microglial polarization. Evidence has suggested that the inhibition of overactivated inflammatory M1 microglia by switching to the protective M2 phenotype might represent a novel therapeutic strategy for mitigating epileptogenesis [1, 19]. It has been reported that intraperitoneal injection of IL-4/ interferon (IFN)-γ modulated the proportions of microglial phenotypes and improved epilepsy outcomes in a pilocarpine model of acquired epilepsy [19]. Another study also indicated that inhibition of MyD88 signaling shifted microglia polarization from M1 to M2 phenotype and attenuated neuronal apoptosis in the hippocampus of mice after status epilepticus [1]. To our knowledge, this study is the first to report that PDYN overexpression promotes microglia polarization toward M2 phenotype in a rat model of epilepsy. Further in vitro assay showed that dynorphin activation of KOR promotes microglia polarization toward M2 phenotype in LPS-stimulated BV-2 microglial cells. Thus, dynorphin might be an effective therapeutic agent for neurological disorders.

Interestingly, in this study, the KOR-specific antagonist GNTI abrogated the dynorphin-A-mediated promotion of BV-2 microglial polarization to the M2 phenotype (group LPS + Dyn-A versus LPS + Dyn-A + GNTI in Fig. 4c, d), indicating that dynorphin-A promoted BV-2 microglial polarization to the M2 phenotype via KOR activation. However, GNTI did not completely reverse the role of Dyn-A, which suggested that in such context, Dyn-A also regulated microglial polarization in a KOR-independent manner.

We also found that the TLR4/NF-κB pathway was involved in the dynorphin/KOR regulated microglial M2 polarization. TLR4 mediates microglial activation [7] and induces NF-κB activation, which plays a critical role in the activation of the M1 microglial phenotype [25]. Inhibition of the TLR4/NF-κB pathway has been reported to induce polarization of the microglia to the M2 phenotype [3, 26]. Our results from rescue experiments demonstrated that activation of the TLR4/NF-κB pathway by overexpression TLR4 and NF-κB could effectively abrogated the dynorphin-mediated promotion of microglial M2 polarization in LPS-induced BV-2 microglial cells, suggesting that dynorphin induced polarization of the microglia to the M2 phenotype by inhibiting the TLR4/NF-κB pathway.

## Conclusion

In conclusion, our findings demonstrate that dynorphin activation of KOR promotes microglia polarization toward M2 phenotype via TLR4/NF-κB pathway. Inhibition of the pro-inflammatory M1 and promotion of anti-inflammatory M2 phenotype are potentially feasible approaches for controlling neuroinflammation. Therefore, dynorphin-induced polarization of the microglia from the M1 toward the M2 phenotype can provide a new strategy for the treatment of neuroinflammation disorders including epilepsy.

## Data Availability

The datasets used and/or analyzed during the current study are available from the corresponding author on reasonable request.

## Abbreviations

Arg-1: Arginase-1
CCK‐8: Cell Counting Kit‐8
CNS: Central nervous system
DMEM: Dulbecco’s modified Eagle's medium
Dyn-A: Dynorphin-A
ELISA: Enzyme-linked immunosorbent assay
FBS: Fetal bovine serum
i.p.: Intraperitoneally
Iba-1: Ionized calcium binding adaptor molecule-1
IFN: Interferon
IL: Interleukin
iNOS: Inducible NO synthase
KOR: Kappa opioid receptor
LPS: Lipopolysaccharide
MR/CD206: Mannose receptor
MyD88: Myeloid differentiation factor 88
NF-κB: Nuclear factor-kappa B
PBS: Phosphate-buffered saline
PC2: Proprotein convertase 2
PDYN: Prodynorphin
qRT-PCR: Quantitative real-time PCR
SD: Standard deviation
TLR4: Toll-like receptor 4
TNF: Tumor necrosis factor
TRIF: tIR-domain-containing adapter-inducing interferon-β
TUNEL: tdT-mediated dUTP Nick-End Labeling
